# Depressive symptoms and associated factors among older patients with arthritis: evidence from a community-based study in eastern China

**DOI:** 10.3389/fpubh.2024.1375106

**Published:** 2024-05-17

**Authors:** Xinyi Wang, Tao Zhang, Xue Gu, Le Xu, Fudong Li, Yujia Zhai, Mengna Wu, Junfen Lin

**Affiliations:** Department of Public Health Surveillance and Advisory, Zhejiang Provincial Center for Disease Control and Prevention, Hangzhou, China

**Keywords:** depressive symptoms, arthritis, older adults, prevalence, factors

## Abstract

**Introduction:**

Depressive symptoms are often experienced by patients with arthritis and are correlated with poor health outcomes. However, the association between depressive symptoms and multidimensional factors (sociodemographic characteristics, health conditions, health behaviors, and social support) among older patients with arthritis in China remains poorly understood. This study aimed to explore the prevalence of depressive symptoms in older patients with arthritis in eastern China and identify the associated factors.

**Methods:**

We analyzed data of 1,081 older patients with arthritis using secondary data from 2014 to 2020 from a community-based ongoing study initiated in 2014 in eastern China. The prevalence of depressive symptoms was calculated, and univariate and multilevel logistic regression analyses were used to identify the associated factors.

**Results:**

The mean age of older patients with arthritis was 69.16 ± 7.13 years; 42.92% were men and 57.08% were women. The prevalence of depressive symptoms in older patients with arthritis was 14.99% (95% confidence interval: 12.91–17.26%), about 1.8 times higher than that in older adults without arthritis (8.49%, *p* < 0.001). Multilevel logistic regression identified perception of poor economic status (odds ratio [OR] = 5.52, *p* < 0.001), multimorbidity (OR = 1.96, *p* = 0.001), limitations in activities of daily living (OR = 2.36, *p* = 0.004), and living alone (OR = 3.13, *p* = 0.026) as factors positively associated with depressive symptoms. Patients diagnosed with arthritis at an older age had lower odds of experiencing depressive symptoms (OR = 0.67, *p* = 0.046).

**Conclusion:**

Screening for depressive symptoms is essential among older patients with arthritis, especially those who perceive themselves as having a poor economic status, are diagnosed at an earlier age, have multimorbidity, have limitations in activities of daily living, and live alone. The associations of age at arthritis diagnosis and dietary behaviors with depressive symptoms require further research.

## Introduction

Arthritis, which causes joint pain and stiffness, affects older adults disproportionately. In China, the prevalence of arthritis among older adults ranged from 24.8–26.6% in 2010 (1), reaching 44.7% in 2018 (2). In the United States, the prevalence of arthritis in adults was approximately 22.7% in 2010–2012 and is projected to rise to 49% by 2040, with the most significant increase occurring in the 65 years and older age group (3). Globally, the number of patients with osteoarthritis (OA), a common type of arthritis, reached 527 million in 2019, with most cases in the 60–64 year age group and age-standardized prevalence peaking in the oldest age group (4). The prevalence of arthritis in older individuals makes arthritis a greater public health challenge in countries with aging populations.

Depressive symptoms are often experienced by patients with arthritis. From 2011 to 2015, the China Health and Retirement Longitudinal Study revealed that 38.9–46.9% of older patients with arthritis reported depressive symptoms using the 10-item Center for Epidemiological Studies Depression Scale (CESD-10) (5). A recent meta-analysis estimated that 41% (95% confidence interval: 32–51%) of patients with rheumatoid arthritis (RA) experienced depression (6). Depressive symptoms have been correlated with a higher risk of adverse health outcomes in patients with arthritis, making screening for and early recognition of them vital. In the United States, patients with arthritis and self-reported doctor-diagnosed depression were 1.7 times more likely to experience poor general health than patients with arthritis but no depression (7). A meta-analysis including studies in several western countries revealed that depressive symptoms increased the risk of all-cause mortality in patients with RA by 80% (8). Evidence from older individuals in China suggests a similar association between depressive symptoms and all-cause mortality in older patients with arthritis (9–11), though limited research was conducted in this specific population.

Chinese studies have explored different factors related to depressive symptoms in patients with arthritis. Mobility disability has been associated with subsequent depressive symptoms as defined by the CESD-10 among middle-aged and older patients with arthritis in China (5). A small sample of community-dwelling older patients with OA in North China showed that Geriatric Depression Scale scores were positively associated with physical function, lower extremity strength, and social support (12). Additional results have been obtained from studies performed in clinical settings. In eastern China, a multicenter study found higher education, functional disability, disease activity, pain, sleep, and fatigue to be related to depressive symptoms, as measured using the Hospital Anxiety and Depression Scale in patients with RA (13–15). In northwestern China, disease duration, disease activity, and low serum 25-hydroxyvitamin D3 levels in patients with RA were associated with depression severity, as assessed using the Hamilton Depression Scale (16). In southern China, patients with RA and lower incomes, no employment, higher disease activity, joint deformities, glucocorticoid treatment, and inflammatory markers were more likely to have depressive symptoms, as defined by the Hamilton Depression Scale (17). For patients with psoriatic arthritis, disease activity and physical function impairment were closely related to depressive symptoms, as evaluated by the Hospital Anxiety and Depression Scale in southern China (18).

To date, most studies on depressive symptoms in older patients with arthritis in China have involved small sample sizes, clinical settings, and a focus on clinical-related factors. The association between depressive symptoms and multidimensional factors, including sociodemographic characteristics, health conditions, health behaviors, and social support, remain poorly understood. Few studies have evaluated the impact of health conditions such as age at diagnosis of arthritis, history of stroke, body mass index (BMI), blood pressure control, and dietary behaviors on depressive symptoms, and few studies have included community-dwelling older adults. This study therefore investigated the association of various factors, including sociodemographic characteristics, health conditions, health behaviors, and social support with depressive symptoms in community-dwelling older patients with arthritis, with the aim of identifying screening indicators for depressive symptoms in this population.

## Methods

### Data source

This cross-sectional study used secondary data from 2014 to 2020 from an ongoing community-based study of aging and health in Zhejiang Province, eastern China. The program was initiated in 2014 and included 11 randomly selected counties/districts out of a total of 90: Tongxiang, Haishu, Changshan, Jingning, Haiyan, Yuhuan, Yuecheng, Yiwu, Binjiang, Nanxun, and Putuo. For each county/district, between one and three towns/sub-districts were randomly selected according to their population density. Several villages/neighborhoods were randomly selected for each town/sub-district. All residents of the selected villages/neighborhoods who met the following inclusion criteria were invited to participate in the study: (1) permanent residents aged 60 years and over, and (2) lived in the area for at least 6 months of the previous year. Multiple individuals from the same household could participate if they each met the inclusion criteria. At least 1,500 participants were randomly recruited in 10 counties/districts; 600 were enrolled in the remaining district because of the sparse population. Seven counties/districts completed the baseline survey in 2014–2015 and four in 2018. New older adults who met the inclusion criteria were invited each year and completed the baseline survey.

The baseline survey was conducted by trained public health practitioners and nurses and involved face-to-face interviews with each participant. Written informed consent was obtained from all participants. Individuals who were unable to read had the informed consent information read to them by their interviewers and they signed with their fingerprints.

By 2020, 21,367 participants had completed the baseline survey. Among the participants, one had missing sex data, eight had missing age data, 23 had missing arthritis data, and 32 had missing depressive symptom data. In total, 40 participants had missing data; 21,327 participants had complete data. Participants ranged in age from 60 to 100 years, with a mean age of 68.21 years; 47.12% were men and 52.88% were women. Compared to participants with incomplete data, those with complete information tended to be younger, married, and working (*p* < 0.05). No significant differences were observed in sex, ethnicity, or perception of economic status (*p* > 0.05). A total of 1,081 participants with self-reported doctor-diagnosed arthritis were included in this study.

### Measures

#### Depressive symptoms

Depressive symptoms were evaluated using the Chinese version of Patient Health Questionnaire-9 (PHQ-9). The PHQ-9 is a brief but well-validated assessment that can be used to screen depression or measure the severity of depressive symptoms (19–21). The Chinese version is widely applied in the Chinese population and examined to have sufficient reliability and validity (22). Participants were asked, “Over the last two weeks, how often have you been bothered by any of the following problems?” Nine problems were provided, such as interest in doing things, depressed feelings, sleep, appetite, and suicidal ideation. The response options were categorized as: (1) not at all (0 score), (2) several days (1 score), (3) more than half the days (2 scores), and (4) nearly every day (3 scores). The PHQ-9 score ranges from 0 to 27, with cutoff scores of 5, 10, 15, and 20 representing mild, moderate, moderately severe, and severe depressive symptoms (also called mild, moderate, moderately severe, and severe depression), respectively (20, 23). In this study, participants with a score of 5 and above were defined as having depressive symptoms.

#### Sociodemographic factors

Sex, age, ethnicity, educational level, marital status, employment status, and perception of economic status were included as sociodemographic factors. Perception of economic status was self-reported.

#### Health conditions and health behaviors

Age at arthritis diagnosis, arthritis duration, multimorbidity, history of stroke, BMI, blood pressure control, cognitive impairment, and limitations in activities of daily living were included as health conditions. Multimorbidity (24) was defined as the presence of two or more of the following conditions: hypertension, diabetes, hyperlipidemia, coronary heart disease, emphysema, asthma, pulmonary tuberculosis, chronic bronchitis, chronic hepatitis, gallstones, nephritis, Parkinson’s disease, tumor, cataract, and glaucoma. Blood pressure below 140/90 mmHg and a BMI of 18.5–24 kg/m^2^ were considered controlled. Cognitive function was examined using the Mini-Mental State Examination, which has a maximum score of 30. Participants who met one of the following criteria were defined as cognitively impaired: (1) illiterate and scored less than 18; (2) educated to primary school level and scored less than 21; and (3) educated to middle school level and above and scored less than 25. Activities of daily living included eating, bathing, dressing, going to the toilet (including incontinence), and functional mobility. Participants who reported being unable to do any of these independently were regarded as having limitations in activities of daily living.

Smoking, alcohol consumption, tea consumption, physical activity, fruit and vegetable consumption, and a sedentary lifestyle were included as health behaviors. Participants were asked about their behaviors in the previous year. Smoking and alcohol consumption were categorized into three groups: (1) never, (2) former, and (3) current. Physical activity and tea consumption were both binary variables with possible answers of yes and no. Vegetable consumption was categorized as: (1) every day and (2) not every day. Fresh fruit consumption was categorized as: (1) every day, (2) every week but not every day, and (3) less than every week. A sedentary lifestyle was identified by asking the question, “How many hours do you spend sitting or lying down each day in addition to sleep?”

#### Social support

Having a child, eating alone, and living alone were included as social support factors. Participants were asked, “Do you currently have a child?” Never having had a child or having a child who had passed away was recorded as a no. Questions on living and eating alone referred to the previous 6 months.

### Statistical analysis

Sociodemographic characteristics, health conditions, health behaviors, and social support factors were examined in terms of their univariate associations with depressive symptoms. The chi-squared test, t-test, and chi-square test for trend were used for univariate analysis, as appropriate. Exact *p*-values were calculated when necessary. Sex, age, education, and variables showing a statistical association with depressive symptoms in the univariate analyses were further included in the multivariate analysis. Multilevel logistic regression with random intercepts was performed to examine factors associated with depressive symptoms in patients with arthritis. The county/district was treated as a group variable in the regression model, considering the area differences in depressive symptoms.

All analyses were performed using STATA software version 16 (StataCorp LLC, College Station, TX, United States). All statistical tests were two-sided. Statistical significance was set at *p* < 0.05.

## Results

### Prevalence of depressive symptoms

The mean age of older patients with arthritis was 69.16 ± 7.13 years, with a gender ratio of 0.75:1 (men:women). Depressive symptoms were experienced by 14.99% (95% confidence interval: 12.91–17.26%) of these individuals, a significantly greater proportion than that of older individuals without arthritis (8.49%, *p* < 0.001) in the 11 selected counties/districts of Zhejiang Province.

### Sociodemographic characteristics of older patients with arthritis

The sociodemographic characteristics and their univariate associations with depressive symptoms are shown in Table 1. A greater proportion of participants with depressive symptoms than those without depressive symptoms were women (68.52% vs. 55.06%, *p* = 0.001). Individuals with depressive symptoms were more likely to be illiterate (58.75% vs. 46.93%, *p* = 0.006), single/widowed/divorced (27.78% vs. 18.72%, *p* = 0.008), and self-report as having a poor economic status (19.75% vs. 5.66%, *p* < 0.001) than those without depressive symptoms. No statistical difference was found in age, ethnicity, or employment status between patients with arthritis with and without depressive symptoms (*p* > 0.05).

**Table 1:** Factors of sociodemographic characteristics and their univariate associations with depressive symptoms.

Sociodemographic characteristics	Total	Non-depressed	Depressed	X^2^	*p*
	(*n* = 1,081)	(*n* = 919)	(*n* = 162)		
Sex, n (%)					
Men	464 (42.92)	413 (44.94)	51 (31.48)	10.18	0.001^**^
Women	617 (57.08)	506 (55.06)	111 (68.52)		
Age, years, n (%)					
60–64	353 (32.65)	302 (32.86)	51 (31.48)	3.24	0.519
65–69	264 (24.42)	226 (24.59)	38 (23.46)		
70–74	214 (19.80)	181 (19.70)	33 (20.37)		
75–79	142 (13.14)	124 (13.49)	18 (11.11)		
> = 80	108 (9.99)	86 (9.36)	22 (13.58)		
Ethnicity^a,b^, n (%)					
Han ethnicity	1,055 (98.51)	900 (98.79)	155 (96.88)	/	0.076
Minorities	16 (1.49)	11 (1.21)	5 (3.13)		
Educational level^a^, n (%)					
Illiterate	522 (48.69)	428 (46.93)	94 (58.75)	7.61	0.006^**^
Literate	550 (51.31)	484 (53.07)	66 (41.25)		
Marital status, n (%)					
Single/Widowed/Divorced	217 (20.07)	172 (18.72)	45 (27.78)	7.05	0.008^**^
Married	864 (79.93)	747 (81.28)	117 (72.22)		
Employment status, n (%)					
Working	356 (32.93)	307 (33.41)	49 (30.25)	1.51	0.469
Retired	483 (44.68)	412 (44.83)	71 (43.83)		
Never work	242 (22.39)	200 (21.76)	42 (25.93)		
Perception of economic status^a^, n (%)					
Rich	206 (19.07)	190 (20.70)	16 (9.88)	43.79	<0.001^***^
Moderate	790 (73.15)	676 (73.64)	114 (70.37)		
Poor	84 (7.78)	52 (5.66)	32 (19.75)		

^a^Missing data observed. ^b^Fisher’s exact test applied. ^**^p < 0.01, ^***^p < 0.001.

### Health conditions and health behaviors of older patients with arthritis

Health conditions and health behaviors and their univariate associations with depressive symptoms are shown in Table 2. Participants with depressive symptoms tended to be diagnosed with arthritis earlier (*p* = 0.022) and had a longer duration of arthritis (*p* = 0.041) than those without depressive symptoms. Participants with depressive symptoms were more likely to have multimorbidity (43.21% vs. 29.27%, *p* < 0.001), cognitive impairment (19.14% vs. 12.40%, *p* = 0.020), and limitations in activities of daily living (16.05% vs. 6.31%, p < 0.001) than those without depressive symptoms. Significant differences were observed in smoking (*p* = 0.037) and alcohol consumption (*p* = 0.009) between participants with and without depressive symptoms. Participants with depressive symptoms were less likely to be tea drinkers (18.52% vs. 30.25%, *p* = 0.002) and were more sedentary (3.09 vs. 2.64 h/day, *p* = 0.007) than those without. No significant differences in the history of stroke, BMI, blood pressure control, physical activity, vegetable consumption, or fruit consumption were observed between patients with arthritis with and without depressive symptoms (*p* > 0.05).

**Table 2:** Health conditions and health behaviors and their univariate associations with depressive symptoms.

Health conditions and health behaviors	Total	Non-depressed	Depressed	X^2^/T	*p*
	(*n* = 1,081)	(*n* = 919)	(*n* = 162)		
Age at arthritis diagnosis^a,b^, years, n (%)					
<30	55 (5.12)	41 (4.49)	14 (8.70)	5.29	0.022^*^
30–39	55 (5.12)	45 (4.92)	10 (6.21)		
40–49	103 (9.58)	85 (9.3)	18 (11.18)		
50–59	324 (30.14)	279 (30.53)	45 (27.95)		
> = 60	538 (50.05)	464 (50.77)	74 (45.96)		
Arthritis duration^a,b^, years, n(%)					
<10	530 (49.30)	454 (49.67)	76 (47.20)	4.17	0.041^*^
10–19	318 (29.58)	277 (30.31)	41 (25.47)		
20–29	114 (10.60)	96 (10.50)	18 (11.18)		
30–39	56 (5.21)	43 (4.70)	13 (8.07)		
> = 40	57 (5.30)	44 (4.81)	13 (8.07)		
History of stroke^a^, n (%)					
No	1,038 (96.47)	885 (96.72)	153 (95.03)	1.15	0.284
Yes	38 (3.53)	30 (3.28)	8 (4.97)		
Multimorbidity, n (%)					
No	742 (68.64)	650 (70.73)	92 (56.79)	12.43	<0.001^***^
Yes	339 (31.36)	269 (29.27)	70 (43.21)		
BMI control^a^, n (%)					
No	503 (47.41)	420 (46.36)	83 (53.55)	2.74	0.098
Yes	558 (52.59)	486 (53.64)	72 (46.45)		
Blood pressure control^a^, n (%)					
No	369 (34.71)	319 (35.17)	50 (32.05)	0.57	0.450
Yes	694 (65.29)	588 (64.83)	106 (67.95)		
Cognitive impairment, n (%)					
No	936 (86.59)	805 (87.60)	131 (80.86)	5.37	0.020^*^
Yes	145 (13.41)	114 (12.40)	31 (19.14)		
Limitations in activities of daily living, n (%)					
No	997 (92.23)	861 (93.69)	136 (83.95)	18.22	<0.001^***^
Yes	84 (7.77)	58 (6.31)	26 (16.05)		
Smoking, n (%)					
Never	778 (71.97)	649 (70.62)	129 (79.63)	6.59	0.037^*^
Former	101 (9.34)	87 (9.47)	14 (8.64)		
Current	202 (18.69)	183 (19.91)	19 (11.73)		
Alcohol consumption, n (%)					
Never	719 (66.51)	602 (65.51)	117 (72.22)	9.46	0.009^**^
Former	85 (7.86)	67 (7.29)	18 (11.11)		
Current	277 (25.62)	250 (27.20)	27 (16.67)		
Physical activity, n (%)					
No	820 (75.86)	697 (75.84)	123 (75.93)	<0.01	0.982
Yes	261 (24.14)	222 (24.16)	39 (24.07)		
Tea consumption, n (%)					
No	773 (71.51)	641 (69.75)	132 (81.48)	9.30	0.002^**^
Yes	308 (28.49)	278 (30.25)	30 (18.52)		
Vegetable consumption^a^, n (%)					
Every day	941 (87.13)	807 (87.91)	134 (82.72)	3.31	0.069
Not every day	139 (12.87)	111 (12.09)	28 (17.28)		
Fruit consumption^a^, n (%)					
Every day	224 (20.74)	191 (20.81)	33 (20.37)	3.21	0.201
Every week but not every day	512 (47.41)	444 (48.37)	68 (41.98)		
Less than every week	344 (31.85)	283 (30.83)	61 (37.65)		
Sedentary lifestyle^a,c^, h/day, mean (SD)	2.71 (1.96)	2.64 (1.89)	3.09 (2.30)	−2.71	0.007^**^

^a^Missing data observed. ^b^Chi-square test for trend applied. ^c^T test applied. ^*^p < 0.05, ^**^p < 0.01, ^***^p < 0.001.

### Social support for older patients with arthritis

Social support factors and their univariate associations with depressive symptoms are presented in Table 3. There was a significantly greater proportion of eating alone (21.60% vs. 11.21%, *p* < 0.001) and living alone (20.37% vs. 9.58%, *p* < 0.001) among participants with depressive symptoms than among those without. Having a child did not significantly affect the prevalence of depressive symptoms (*p* < 0.05).

**Table 3:** Social support and their univariate associations with depressive symptoms.

Social support	Total	Non-depressed	Depressed	X^2^	*p*
	(*n* = 1,081)	(*n* = 919)	(*n* = 162)		
Having a child^a^, n (%)					
No	16 (1.48)	16 (1.74)	0 (0.00)	/	0.150
Yes	1,065 (98.52)	903 (98.26)	162 (100.00)		
Eating alone, n (%)					
No	943 (87.23)	816 (88.79)	127 (78.40)	13.37	<0.001^***^
Yes	138 (12.77)	103 (11.21)	35 (21.60)		
Living alone, n (%)					
No	960 (88.81)	831 (90.42)	129 (79.63)	16.14	<0.001^***^
Yes	121 (11.19)	88 (9.58)	33 (20.37)		

^a^Fisher’s exact test applied. ^***^p < 0.001.

### Multilevel logistic regression for depressive symptoms

Sex, age, education level, marital status, perception of economic status, age at diagnosis of arthritis, arthritis duration, multimorbidity, cognitive impairment, limitations in activities of daily living, smoking, alcohol consumption, tea consumption, sedentary hours, living alone, and eating alone were further included in a multilevel logistic regression analysis. Associations between the selected factors and depressive symptoms are shown in Table 4. The perception of poor economic status showed a strong statistical association with depressive symptoms compared to the perception of a high economic status (odds ratio [OR] = 5.52, *p* < 0.001). Age at arthritis diagnosis was negatively associated with depressive symptoms, with a later diagnosis corresponding to lower odds of depressive symptoms (OR = 0.67, *p* = 0.046). Multimorbidity (OR = 1.96, *p* = 0.001), limitations in activities of daily living (OR = 2.36, *p* = 0.004), and living alone (OR = 3.13, *p* = 0.026) were all significantly associated with depressive symptoms. All remaining variables failed to reach statistical significance (*p* > 0.05).

**Table 4:** Multilevel logistic regression for the association between the selected factors and depressive symptoms.

Factors	OR (95% Confidence Interval)	*p*
Sex		
Men	1.00	
Women	1.46 (0.84, 2.53)	0.177
Age		
60–64	1.00	
65–69	1.09 (0.65, 1.83)	0.748
70–74	1.34 (0.71, 2.51)	0.365
75–79	0.87 (0.41, 1.83)	0.707
> = 80	1.28 (0.55, 2.99)	0.565
Educational level		
Illiterate	1.00	
Literate	0.71 (0.47, 1.07)	0.102
Marrital status		
Single/Widowed/Divorced	1.00	
Married	1.16 (0.67, 2.01)	0.596
Perception of economic status		
Rich	1.00	
Moderate	1.61 (0.87, 2.95)	0.127
Poor	5.52 (2.57, 11.87)	<0.001^***^
Age at arthritis diagnosis (years)	0.67 (0.45, 0.99)	0.046^*^
Arthritis duration (years)	0.76 (0.52, 1.10)	0.150
Multimorbidity		
No	1.00	
Yes	1.96 (1.33, 2.88)	0.001^**^
Cognitive impairment		
No	1.00	
Yes	1.51 (0.91, 2.50)	0.111
Limitations in activities of daily living		
No	1.00	
Yes	2.36 (1.31, 4.25)	0.004^**^
Smoking		
Never	1.00	
Former	1.13 (0.51, 2.53)	0.765
Current	1.06 (0.53, 2.1)	0.869
Alcohol consumption		
Never	1.00	
Former	1.42 (0.73, 2.74)	0.301
Current	0.86 (0.51, 1.46)	0.580
Tea consumption		
No	1.00	
Yes	0.69 (0.42, 1.14)	0.148
Sedentary lifestyle (h/day)	1.08 (0.99, 1.18)	0.072
Eating alone		
No	1.00	
Yes	0.70 (0.26, 1.91)	0.492
Living alone		
No	1.00	
Yes	3.13 (1.14, 8.54)	0.026^*^

^*^p < 0.05, ^**^p < 0.01, ^***^p < 0.001.

## Discussion

### Prevalence of depressive symptoms in patients with arthritis

The prevalence of depressive symptoms, although high in patients with arthritis, varies according to the type of arthritis, method of assessing depressive symptoms, and population studied (25). A meta-analysis published in 2020 revealed that patients with RA had a 47% higher risk of depression than older adults without RA (8). A separate meta-analysis in the same year showed that 17% of individuals with psoriatic arthritis experienced depression, 1.68 times higher than the proportion of those without psoriatic arthritis (26). A meta-analysis in 2016 estimated that the pooled prevalence of depressive symptoms was 19.9% among patients with OA, with a relative risk of 1.17 compared to individuals without OA (27). When including all forms of arthritis, the national prevalence of depressive symptoms classified based on a frequency question and an intensity question of depressed feeling in the United States between 2015 and 2017 was 12.1% among adults with arthritis and 4.7% among those without arthritis (28). The present study on community-dwelling older adults revealed a prevalence of depressive symptoms of nearly 15.0% in patients with arthritis, approximately 1.8 times higher than in those without arthritis. A similar gap was observed in a longitudinal study on older Chinese patients with arthritis (29). Evidence has therefore consistently demonstrated that patients with arthritis experience a more significant burden of depressive symptoms than their arthritis-free counterparts.

### Factors associated with depressive symptoms in patients with arthritis

The current study identified perception of economic status, multimorbidity, limitations in activities of daily living, living alone, and age at arthritis diagnosis as factors significantly associated with depressive symptoms in older patients with arthritis.

Previous studies have also associated poor economic status with depression, as evaluated by the Hamilton Depression Scale-17 and PHQ-9 in patients with arthritis (17, 30). The relationship between economic status and depressive symptoms can be partly explained by arthritis-related costs, which are more difficult to bear for those with lower incomes, as indicated by a qualitative study performed in Australia (31). In addition, depressive symptoms as assessed using the CESD-10 have been associated with higher healthcare costs and productivity loss among Chinese individuals with 12 chronic diseases (32), which may impact economic status. Furthermore, lower socioeconomic status has been related to multimorbidity patterns of cardiovascular and metabolic disorders (33), indicating that economic conditions may affect depressive symptoms partly through multimorbidity.

The present study revealed that multimorbidity increased the odds of depressive symptoms among older patients with arthritis nearly two-fold. Our results are consistent with those of two previous studies. The United States National Health Interview Survey (2015–2017) found that the prevalence of depressive symptoms classified based on a frequency question and an intensity question of depressed feeling in patients with arthritis and more than two chronic conditions was double that of patients with one or two conditions (28). The Korea National Health and Nutrition Examination Survey revealed that patients with RA with three or more comorbidities had five-fold higher odds of depression as measured by the PHQ-9 than those without (34). In addition to its relationship with depressive symptoms, multimorbidity has been found to be related to limitations in activities of daily living in China, India, and the United States (35–37). This suggests that multimorbidity may be indirectly linked to depressive symptoms through functional limitations.

Our results associating limitations in activities of daily living with depressive symptoms in older patients with arthritis is consistent with the findings of many previous studies. A study in Korea found that patients with RA were three times more likely to experience depression, as assessed by the PHQ-9, if they also had limitations in activities of daily living (34). A study in Taiwan Province of China also associated higher functional ability with lower levels of depression measured by the Geriatric Depression Scale (38). A longitudinal study conducted in China revealed a reciprocal relationship between depressive symptoms, defined by the CESD-10, and mobility disability in patients with arthritis (5), reflecting their complex interaction. Depressive symptoms, assessed using the Geriatric Depression Scale, can lead to functional limitations by impairing mobility capacities and exacerbating feelings of pain (39). In addition, limitations in activities of daily living can reduce independence and participation in social activities, thus increasing the risk of depressive symptoms (40).

In the current study, living alone was associated with depressive symptoms, as expected. Living alone can result in limited social and emotional support, and the latter was seen among patients with arthritis in relation to depression assessed by a modified version of the CESD in the United States (41) and the Beck Depression Inventory II in Japan (42). It has also been reported that limitations in activities of daily living were significantly more prevalent in older Korean adults living alone than in those living with relatives (43). People living alone might be more affected by limitations in activities of daily living because they receive less help from others. Therefore, we speculate that living alone has a greater influence on depressive symptoms in patients with arthritis and limitations in activities of daily living than in those without.

We found that arthritis diagnosed at an earlier age was significantly associated with depressive symptoms, even after controlling for disease duration. Age at diagnosis provides information on disease duration and reflects the impact of the disease at different life stages. It may be an important screening factor for depressive symptoms in patients with arthritis; however, supporting evidence is limited. There are several possible explanations for this finding. First, arthritis is debilitating and influences the ability of patients to work, thus affecting their economic status. Individuals diagnosed at an earlier age would be more affected by this. Second, patients with early-onset arthritis have a longer interval between disease onset and diagnosis (44), leading to negative psychological effects. Third, patients with late-onset arthritis may be better emotionally and mentally equipped to cope with the disease because they may already have comorbidities (44) and therefore have experience coping with illness. Fourth, patients of different ages at diagnosis are expected to have different clinical characteristics and thus depressive symptoms. Previous studies have reported conflicting results based on different indicators. Radiographic damage progression and time to remission did not differ between patients with early- and late-onset RA (45, 46). However, patients with late-onset RA had greater inflammation and bone erosion (47–49). Further large-scale research on socioeconomic and clinical factors is needed to examine these associations.

Smoking and alcohol consumption were not associated with depressive symptoms in our study, consistent with a recent meta-analysis of 11 studies involving older Chinese adults (40). Physical activity has been shown to be inversely associated with depressive symptoms as determined by the CESD among Chinese individuals, such as older veterans (50), middle-aged and older city dwellers (51), and older women (52). However, the current study did not observe such an association among older patients with arthritis. This may be due to different target populations and physical activity evaluations. According to the assessment of CESD-10, the association between physical activity and depressive symptoms depends on the intensity of the physical activity (53, 54). The pain and functional limitations experienced by patients with arthritis may not allow for activity of sufficient intensity to combat depressive symptoms.

In contrast to previous studies on the general population (55, 56), vegetable and fruit consumption were not associated with depressive symptoms in the present study. This may be due to the different types of vegetable and fruit consumed and the frequency of consumption. A study in Korea found an association between physician diagnosed depression and a high consumption of green vegetables and fruits, whereas no association was observed when the consumption of vegetables or fruit was moderate (57). A study in Japan found an association between tomato consumption and depressive symptoms assessed by the Zung Self-Rating Depression Scale, but not the consumption of any other vegetables (58). Further studies are required to determine the types and frequencies of vegetable and fruit consumption associated with depressive symptoms.

Tea consumption has previously been associated with lower odds of depressive symptoms, as assessed by the CESD-10 and Geriatric Depression Scale, among older Chinese adults (59, 60). The antidepressant effects of tea can be partially attributed to its antioxidant and anti-inflammatory constituents (59). The social aspects of tea drinking may also contribute to this effect (60, 61). However, tea consumption was not related to depressive symptoms in the present study. Regular tea consumption may be insufficient for its biomedical effect on depressive symptoms in patients with arthritis who are in a state of inflammation. Patients with arthritis may also have fewer opportunities for social tea consumption, as they are more likely to have functional disabilities.

### Practical and clinical implications of the findings

The present study revealed a more significant burden of depressive symptoms in older adults with arthritis than their arthritis-free counterparts. It highlights the importance of recognizing and addressing the mental health needs of older patients with arthritis, which could further improve their health outcomes.

Our findings identified multidimensional associated factors of depressive symptoms among older patients with arthritis, including poor economic status, age at arthritis diagnosis, multimorbidity, limitations in activities of daily living, and living alone. These factors can be developed into a screening protocol for depressive symptoms and applied in both routine clinical arthritis care and community-based programs, making it possible for healthcare providers to prioritize resources and support for those who might be most likely to experience depressive symptoms. Furthermore, the inverse relationship between age at arthritis diagnosis and depressive symptoms observed in the study suggests that early intervention may play a crucial role in the management of mental health in older patients with arthritis. This could involve developing educational materials and support groups specifically for newly diagnosed patients aimed at helping them deal with depressive symptoms that might occur.

In conclusion, this study provides valuable insights into public health practice and clinical care for older patients with arthritis. By recognizing the heavy burden of depressive symptoms among older patients with arthritis and the associated multidimensional factors, healthcare systems could develop more comprehensive strategies to better support this vulnerable population, improving not only their physical health but also their mental well-being.

### Strengths and limitations

Our data were collected from a community-dwelling population and should therefore have better generalizability than previous studies performed in clinical settings. Many factors were included in the analysis, offering a more comprehensive picture of depressive symptoms in patients with arthritis. Nevertheless, our study has certain limitations. First, we did not examine the temporal association between arthritis and depressive symptoms. However, emerging evidence indicates that their correlation is probably bidirectional, as they impact each other and share mechanisms such as inflammation (62). The factors identified in the present study could serve as screening indicators for depressive symptoms among patients with arthritis in the community and play an important role in identifying those who require additional mental health care. Second, the presence of arthritis was determined by asking patients whether they had been previously diagnosed; verification of the diagnosis of arthritis was not performed, and misclassification of arthritis status may have occurred. A nationally representative study in older Chinese adults found that the prevalence of self-reported doctor-diagnosed arthritis was close to that of symptom-based diagnosed arthritis, with the prevalence of undiagnosed arthritis approximately 5% (63). Therefore, the potential number of misclassified cases, if any, is believed to be low. Third, disease intensity, such as disease activity and pain (15, 64, 65), have previously been shown to be associated with depressive symptoms in patients with arthritis; however, this information was not available in our study. Therefore, their association with depressive symptoms could not be analyzed. Fourth, treatment has also been suggested to be related to depressive symptoms among patients with arthritis (66), but was not included in the current analysis. The prevalence of untreated arthritis was estimated by a large-scale study of older Chinese individuals in 2007–2010 to be over 30% (63). Missing therapy data should be considered when interpreting these results. However, the prevalence of treated but uncontrolled arthritis has been reported to be up to 77% in older patients in six middle-income countries, including China (67), indicating unsatisfactory treatment outcomes of patients with arthritis. Therefore, the supposed benefits of arthritis therapy on depressive symptoms may be affected by its low efficacy.

## Conclusion

The present study found that the prevalence of depressive symptoms was approximately 1.8 times higher in older adults with arthritis than in those without arthritis. Screening for depressive symptoms is essential among older patients with arthritis, especially those who perceive themselves as economically poor, are diagnosed with arthritis at an earlier age, exhibit multimorbidity, have limitations in activities of daily living, and live alone. The associations of age at arthritis diagnosis and dietary behaviors with depressive symptoms require further study in different populations.

## Data availability statement

The raw data supporting the conclusions of this article will be made available by the corresponding author upon reasonable request and with the permission of the Ethics Committee of Zhejiang Provincial Center for Disease Control and Prevention.

## Ethics statement

The studies involving humans were approved by the Ethics Committee of Zhejiang Provincial Center for Disease Control and Prevention. The studies were conducted in accordance with the local legislation and institutional requirements. The participants provided their written informed consent to participate in this study.

## Author contributions

XW: Writing – original draft, Writing – review & editing. TZ: Writing – review & editing. XG: Writing – review & editing. LX: Writing – review & editing. FL: Writing – review & editing. YZ: Writing – review & editing. MW: Writing – review & editing. JL: Writing – review & editing.
